# Hyaluronic Acid as an Adjunctive Therapy in Periodontal and Dental Treatment of Medically Compromised Patients: A Narrative Review

**DOI:** 10.3390/jfb17030154

**Published:** 2026-03-20

**Authors:** Meizi Eliezer, Ruxandra Christodorescu, Alla Belova, Darian Rusu, Stefan Milicescu, Moshe Cohen, Stefan-Ioan Stratul

**Affiliations:** 1 University Clinic of Periodontology, Anton Sculean Research Center for Periodontal and Peri-Implant Diseases, Faculty of Dental Medicine, “Victor Babeș” University of Medicine and Pharmacy, Bulevardul Revoluției No. 9, 300041 Timișoara, Romaniastratul.stefan@umft.ro (S.-I.S.); 2 Department V—Internal Medicine, Research Center—Institute of Cardiovascular Diseases, “Victor Babeș” University of Medicine and Pharmacy, Timișoara, Bulevardul Revoluției No. 12, 300024 Timișoara, Romania; 3 Department of Dental Esthetics, Faculty of Dentistry, “Carol Davila” University of Medicine and Pharmacy, 9 Șoseaua Olteniței, Sector 4, 041292 Bucharest, Romania; 4 Department of Biophysics and Cell Biology, Faculty of Medicine, University of Debrecen, Egyetem ter 1, H-4032 Debrecen, Hungary

**Keywords:** hyaluronic acid, functional biomaterials, periodontal therapy, medically compromised patients, diabetes mellitus, oral wound healing, peri-implant disease, oral mucositis

## Abstract

Hyaluronic acid (HA) is a biologically active glycosaminoglycan with recognized roles in wound healing and inflammation modulation, and its adjunctive use in dental and periodontal therapy has gained interest, particularly in medically compromised patients. This narrative review critically evaluated preclinical and clinical evidence on locally applied HA in periodontal, oral surgical, peri-implant, and oral medicine treatments in patients with systemic conditions. A literature search of PubMed/MEDLINE, Scopus, and Web of Science (January 2015–December 2025) identified in vivo translational studies, randomized and controlled clinical trials, and selected systematic reviews involving medically compromised populations. Qualitative synthesis focused on biological plausibility, clinical outcomes, and safety. Nine core studies were included, comprising two preclinical in vivo investigations and seven clinical trials. In diabetic models, cross-linked high-molecular-weight HA reduced macrophage infiltration and delayed collagen membrane degradation without impairing angiogenesis. Clinically, adjunctive HA use in patients with type 2 diabetes mellitus was associated with modest but statistically significant short-term improvements in clinical attachment level (CAL) and enhanced early soft tissue healing following tooth extraction. In peri-implantitis therapy and oncology-related oral complications, HA application was linked to reduced inflammatory markers, decreased lesion severity, and improved patient-reported symptoms. No systemic adverse effects were reported. Overall, HA appears to be a locally safe adjunct that may support early healing and inflammation control in medically compromised patients, although its effects are primarily short-term and do not indicate disease-modifying potential.

## 1. Introduction

Hyaluronic acid (HA), also known as hyaluronan, is a naturally occurring, non-sulfated glycosaminoglycan and a ubiquitous component of the extracellular matrix of connective tissues. Structurally, HA is a linear polysaccharide composed of repeating disaccharide units of D-glucuronic acid and N-acetyl-D-glucosamine, a configuration that confers high water-binding capacity, viscoelasticity, and space-filling properties [1]. These physicochemical characteristics underpin its role in tissue hydration, mechanical support, and regulation of cell migration and proliferation, thereby contributing to tissue homeostasis [1].

Beyond its structural function, HA acts as a biologically active matrix component involved in inflammation and wound healing. Its biological behavior is strongly influenced by molecular weight: high-molecular-weight (HMW) HA is generally associated with anti-inflammatory and immunomodulatory effects, whereas fragmented low-molecular-weight (LMW) HA has been linked to angiogenesis and pro-inflammatory signaling [2,3]. Advances in biomaterial engineering have enabled the development of chemically modified and cross-linked HMW HA formulations with enhanced resistance to enzymatic degradation and prolonged tissue residence time, expanding its application as a functional biomaterial in regenerative medicine, including periodontal and oral tissue engineering [2,4,5].

HMW HA has been widely used in medical disciplines as an adjunctive biomaterial in conditions characterized by chronic inflammation, impaired angiogenesis, and delayed tissue repair, such as diabetes mellitus, osteoarthritis, chronic skin wounds, and inflammatory disorders [4,6,7,8,9]. Clinical and translational evidence indicates that HA-based interventions can modulate the local inflammatory milieu, promote epithelialization, and support tissue regeneration, while maintaining a favorable safety profile [2,4,6]. In diabetic wound models and clinical studies of diabetic foot ulcers, HMW HA has been associated with improved healing kinetics and reduced wound size, supporting its relevance in compromised healing environments [10,11]. As with other biologically active biomaterials initially validated in medicine, these findings have stimulated growing interest in translating HA-based strategies into dental and periodontal applications [2,5].

Early dental investigations primarily evaluated HA in healthy experimental and clinical models to establish baseline biological effects and local safety. Only more recently has attention shifted toward medically compromised conditions, in which host–biomaterial interactions are fundamentally altered. In this context, disease-specific translational models have provided important insights. Using a streptozotocin-induced uncontrolled diabetic rat model, cross-linked HMW HA (CLHA) was shown to delay collagen membrane degradation and preserve structural integrity under hyperglycemic conditions, without impairing angiogenesis or tissue integration [12,13]. Notably, these effects were observed selectively in diabetic animals and not in normoglycemic controls, suggesting a disease-dependent biomaterial response rather than a nonspecific material effect [12,13,14,15]. Mechanistic analyses further demonstrated reduced macrophage infiltration within CLHA-treated membranes, supporting a local immunomodulatory mode of action in a highly inflammatory systemic environment [13]. These findings highlight an emerging concept in functional biomaterials research: that material performance may depend on the systemic inflammatory and metabolic context of the host.

The bidirectional relationship between systemic health and oral tissue response is well established. Chronic systemic diseases, including diabetes mellitus, malignancies, autoimmune disorders, and osteoporosis, as well as modern pharmacological therapies such as chemotherapy, immunomodulators, antiresorptive agents, and anti-angiogenic drugs, can significantly alter wound healing dynamics and treatment outcomes following dental and periodontal interventions [16,17,18,19]. These challenges are particularly relevant in periodontal therapy, oral surgery, and implant dentistry, where predictable soft and hard tissue regeneration is critical for long-term success. Despite the increasing prevalence of medically complex patients in dental practice, evidence on the adjunctive use of HA-based biomaterials under systemic compromise remains limited, heterogeneous, and occasionally inconsistent [20,21,22].

Given this context, a narrative literature review provides a valuable framework to integrate heterogeneous preclinical and clinical data and to contextualize emerging biomaterial concepts that are not yet amenable to formal systematic analysis. Hence, the aim of the present review is to critically evaluate current preclinical and clinical evidence on the adjunctive use of hyaluronic acid as a functional biomaterial in periodontal, oral surgical, peri-implant, and oral medicine applications involving medically compromised patients. Particular emphasis is placed on biological plausibility, host–material interactions, safety, and clinically relevant outcomes. By synthesizing current evidence, this review seeks to clarify the potential role and current limitations of HA-based biomaterials in compromised healing environments and to identify priorities for future translational and clinical research.

## 2. Materials and Methods

### 2.1. Literature Search Strategy

This narrative review follows the recommendations of the Scale for the Assessment of Narrative Review Articles (SANRA) [23]. A structured, non-systematic literature search was designed to identify relevant preclinical and clinical evidence on the use of hyaluronic acid (HA) as a functional biomaterial in oral and periodontal applications involving medically compromised patients. Electronic searches were conducted in PubMed/MEDLINE, Scopus, and Web of Science, covering publications from January 2015 to December 2025.

The search strategy combined free-text keywords and database-specific indexing terms related to HA, oral biomaterials, and systemic disease, including: “hyaluronic acid,” “hyaluronan,” “periodontal therapy,” “oral surgery,” “dental extraction,” “peri-implantitis,” “oral mucositis,” “diabetes mellitus,” “chemotherapy,” “radiotherapy,” and “systemic disease.” Search terms were adapted as appropriate for each database. In addition, the reference lists of key publications were manually screened to identify relevant articles not retrieved through the electronic search.

### 2.2. Scope of Evidence and Study Selection

The scope of the review encompassed preclinical in vivo studies, randomized controlled clinical trials, controlled clinical studies, and selected systematic reviews evaluating the adjunctive use of locally applied HA-based formulations in periodontal, oral surgical, peri-implant, and oral medicine contexts. Particular emphasis was placed on studies addressing medically compromised conditions or investigating the biological behavior, safety, and host–material interactions of HA under altered or impaired healing environments.

Evidence was selected based on relevance to functional biomaterial performance, biological plausibility, and translational significance rather than on formal risk-of-bias assessment, in accordance with the narrative review design. No quantitative meta-analysis was performed. To enhance transparency and reduce potential selection bias, the identification of the nine core studies followed predefined inclusion and exclusion criteria. The inclusion criteria were defined as: (1) preclinical in vivo studies or controlled clinical trials; (2) investigations involving medically compromised conditions or impaired healing models; and (3) local HA application in oral or periodontal contexts. The exclusion criteria included in vitro-only studies, case reports, studies in systemically healthy populations without compromised models, and non-oral applications.

### 2.3. Data Availability and Ethical Considerations

This article is based exclusively on previously published studies. No new experimental data involving human participants or animals were generated, and therefore no ethical approval was required. All data referenced in this review are available in the original publications cited. No restrictions apply to the availability of materials, data, or protocols discussed.

## 3. Results

The literature search identified two preclinical in vivo animal studies and seven randomized controlled clinical trials evaluating the adjunctive use of hyaluronic acid (HA) in dental and oral applications involving medically compromised patients. An overview of study design, systemic condition, HA formulation, application protocol, principal outcomes, and reported safety findings is summarized in Table 1 (preclinical studies) and Table 2 (clinical studies).

### 3.1. Periodontal Therapy in Diabetes Mellitus

#### 3.1.1. Preclinical Evidence in Diabetic Models

Preclinical studies using streptozotocin-induced uncontrolled diabetic rat models demonstrated altered biomaterial behavior under compromised healing conditions. Diabetes was associated with accelerated collagen membrane degradation compared with normoglycemic controls [14,15]. Immersion of collagen membranes in cross-linked high-molecular-weight hyaluronic acid (CLHA) resulted in preservation of membrane thickness and increased residual collagen area relative to untreated membranes [12]. These effects were observed selectively in diabetic animals, whereas no significant differences were detected in normoglycemic controls, indicating a disease-dependent response [12,14,15] (Table 1).

Histological analyses further showed that CLHA-treated membranes maintained tissue integration and angiogenesis without inducing adverse inflammatory reactions. Quantitative assessments demonstrated significantly reduced CD68^+^ macrophage infiltration within CLHA-treated membranes under diabetic conditions, consistent with delayed membrane resorption [12,13] (Table 1).

#### 3.1.2. Clinical Evidence in Patients with Type 2 Diabetes Mellitus

One randomized controlled clinical trial evaluated the adjunctive application of 0.2% hyaluronic acid as part of non-surgical periodontal therapy in patients with type 2 diabetes mellitus and periodontitis [24] (Table 2). In the HA-treated group, statistically significant reductions in bleeding on probing (BOP) and probing pocket depth (PPD) were observed at 4 weeks, accompanied by a significant improvement in CAL. Comparable intra-group improvements were also observed in the placebo group [24]. Intergroup analysis demonstrated a statistically significant difference favoring HA only for CAL change over time. No statistically significant intergroup differences were observed for PPD or BOP. Both groups demonstrated significant intra-group improvements; however, superiority of HA over placebo was limited to CAL. Gingival crevicular fluid analysis revealed significant reductions in interleukin-1β concentrations in both HA and placebo groups, with no statistically significant intergroup differences. No treatment-related adverse events were reported [24] (Table 2).

### 3.2. Oral Surgery and Extraction Socket Healing

Randomized controlled trials evaluating intra-socket application of HA following tooth extraction demonstrated statistically significant improvements in early soft tissue healing and postoperative outcomes [25,26] (Table 2). In a triple-blind randomized clinical trial, HA-treated extraction sockets exhibited significantly higher radiographic bone density and more advanced trabecular organization at 90 days compared with control sites [25].

Postoperative morbidity outcomes were also affected. In mandibular third molar extraction, HA application was associated with significantly lower pain scores during the first postoperative week compared with chlorhexidine-treated controls. Swelling scores followed a similar trend, although intergroup differences diminished after day 7 [26].

In medically compromised patients with type 2 diabetes mellitus, a randomized split-mouth clinical trial demonstrated significantly faster epithelial closure of HA-treated extraction sockets. At 14 days, complete soft tissue closure was observed in approximately 78–82% of HA-treated sites compared with 45–50% of untreated control sites [26] (Table 2). No statistically significant differences were reported for postoperative infection rates, delayed healing, or adverse events [27].

Cone beam computed tomography (CBCT)-based assessments in selected trials suggested increased early trabecular density and organization at HA-treated sites; however, quantitative differences in ridge width or vertical dimensional stability at follow-up periods of six months or longer were inconsistently reported and did not reach statistical significance [25,27] (Table 2).

### 3.3. Peri-Implant Diseases

Most clinical studies evaluating HA in peri-implant disease were conducted in mixed or non-stratified patient populations, with limited reporting of systemic disease status [28,29] (Table 2).

In a randomized controlled clinical trial evaluating adjunctive use of 0.8% HA gel in peri-implantitis therapy, patients with diabetes, hypertension, and osteoporosis were included. Mean peri-implant probing depth was significantly reduced in the HA-treated group at 45 and 90 days compared with one control group, while comparisons with a second control group did not reach statistical significance [28] (Table 2). BOP decreased over time in all groups, with a greater reduction observed in the HA group, although intergroup differences did not reach statistical significance [28].

Analysis of peri-implant crevicular fluid demonstrated a statistically significant reduction in interleukin-1β concentrations at 45 days in HA-treated implants with baseline probing depths of ≥5 mm compared with controls [28]. A separate randomized clinical trial investigating peri-implantitis reported short-term alterations in the peri-implant subgingival microbiome following adjunctive HA application; however, long-term clinical and radiographic outcome data were limited [29] (Table 2).

### 3.4. Oncology-Related Oral Complications

#### 3.4.1. Radiotherapy-Induced Oral Mucositis (Adults)

In controlled clinical studies involving patients undergoing hematopoietic stem cell transplantation, topical application of sodium hyaluronate-based formulations resulted in statistically significant reductions in oral mucositis severity compared with chlorhexidine-based care (Table 2). HA-treated patients exhibited lower WHO mucositis grades, reduced Oral Mucositis Assessment Scale (OMAS) scores, shorter mucositis duration, and lower pain intensity. No treatment-related systemic adverse events were reported [30] (Table 2).

In a triple-blind randomized clinical trial in patients receiving head and neck radiotherapy (radiation-induced oral mucositis context), a mouthwash containing hyaluronic acid, vitamin E, and triamcinolone resulted in significantly lower mucositis grades and pain scores compared with triamcinolone alone throughout weeks 1–4 of treatment [31] (Table 2).

#### 3.4.2. Chemotherapy-Induced Oral Mucositis (Pediatric Patients)

A randomized clinical trial in pediatric patients comparing an HA-based spray with standard care reported no significant differences in mucositis incidence or severity. However, the duration of mucositis was significantly shorter in the HA-treated group, with a lower proportion of patients exhibiting persistent mucositis at day 7 [32] (Table 2).

#### 3.4.3. Radiotherapy-Induced Xerostomia (Adults)

In a double-blind randomized crossover clinical trial, sodium hyaluronate mouthwash use in radiotherapy-associated xerostomia resulted in significant improvements in xerostomia symptoms compared with placebo. Modified Xerostomia Questionnaire (XQ) scores decreased significantly from baseline, and patient satisfaction scores were significantly higher in the HA group [33] (Table 2).

**Table 1:** Preclinical in vivo studies evaluating hyaluronic acid under compromised healing conditions.

	Study (Year)	Species/Model	Methodological Design	Experimental Setting	HA Formulation	Primary Outcomes	Key Findings	Clinical Relevance
Uncontrolled diabetes mellitus	Eliezer et al., 2019 [12]	Rat	Preclinical in vivo translational study (streptozotocin-induced diabetic model)	GBR CM degradation	Cross-linked HMW HA	Membrane thickness, residual collagen area	CLHA significantly delayed membrane degradation and preserved collagen structure in diabetic animals; no effect observed in normoglycemic controls (*p* < 0.05)	Demonstrates disease-dependent stabilization of biomaterials under hyperglycemic conditions
Uncontrolled diabetes mellitus	Eliezer et al., 2022 [13]	Rat	Preclinical in vivo translational study with mechanistic analysis	GBR CM IHC	Cross-linked HMW HA	CD68^+^ macrophage density, angiogenesis, CM integrity	CLHA significantly reduced macrophage infiltration without impairing angiogenesis or tissue integration (*p* < 0.05)	Supports a local anti-inflammatory mechanism in severely compromised healing environments

Abbreviations: GBR, guided bone regeneration; CM, collagen membrane; IHC, immunohistochemistry; HA, hyaluronic acid; HMW, high-molecular-weight; CLHA, cross-linked hyaluronic acid.

**Table 2:** Clinical in vivo studies evaluating hyaluronic acid under compromised healing conditions.

Systemic Condition	Study (Year)	Methodological Design	Clinical Setting	HA Formulation	Outcome Measure	Time Point	HA Group (Mean ± SD/%)	Control Group (Mean ± SD/%)	Effect Direction of HA	*p*-Value
Type 2 Diabetes Mellitus	Devina et al., 2024 [23]	Randomized controlled clinical trial (parallel group)	Non-surgical periodontal therapy	0.2% non–cross-linked HA gel (topical, local application)	CAL (mm)	4 weeks	4.33 ± 3.16	5.33 ± 2.29	Greater CAL gain	0.042
Type 2 Diabetes Mellitus	Ruggiero et al., 2024 [26]	Randomized split-mouth controlled clinical trial	Extraction socket healing	CLHA gel (intra-socket application)	Complete epithelial closure (%)	14 days	78–82%	45–50%	Faster healing	<0.05
Diabetes/HTN/Osteoporosis	Sánchez-Fernández et al., 2021 [27]	Randomized controlled clinical trial	Peri-implantitis	0.8% HA gel (local peri-implant application)	PPD (mm)	90 days	2.97 ± 0.64	3.62 ± 0.83 (baseline)	↓ PPD	0.01
Diabetes/HTN/Osteoporosis	Sánchez-Fernández et al., 2021 [27]	Randomized controlled clinical trial	Peri-implantitis	0.8% HA gel (local peri-implant application)	IL-1β (pg/mL)	45 days	49 ± 47	100 ± 96	↓ inflammation	0.04
Oncology (radiotherapy)	Agha-Hosseini et al., 2021 [30]	Triple-blind randomized clinical trial	Oral mucositis	HA-containing mouthwash (HA + vitamin E + triamcinolone)	WHO grade/pain	Weeks 1–4	Lower scores	Higher scores	Symptom reduction	<0.001
Oncology (pediatric chemotherapy)	Ghoroubi et al., 2024 [31]	Randomized clinical trial	Oral mucositis	HA-based oral spray	Persistent mucositis (%)	Day 7	25.0%	72.7%	Shorter duration	0.01
Oncology (radiotherapy)	Rupe et al., 2023 [32]	Double-blind randomized crossover clinical trial	Xerostomia	Sodium hyaluronate mouthwash	Modified XQ score	End of treatment	48.4 ± 24.1	65.0 ± 23.6	↓ xerostomia	0.01
Oncology (HSCT)	Ruggiero et al., 2018 [29]	Controlled clinical study (oncology cohort)	Oral mucositis after hematopoietic stem cell transplantation	Sodium Hyaluronate +amino acid Spray (Mucosamin^®^)	WHO/OMAS/VAS	Hospitalization period	Lower mucositis grades, reduced pain and duration	Higher severity and longer duration	↓ severity, ↓ duration, ↓ pain	<0.05

Abbreviations: CAL, clinical attachment level; CLHA, cross-linked hyaluronic acid; HA, hyaluronic acid; HSCT, hematopoietic stem cell transplantation; HTN, hypertension; IL-1β, interleukin-1 beta; OMAS, Oral Mucositis Assessment Scale; PPD, probing pocket depth; SD, standard deviation; VAS, Visual Analog Scale; WHO, World Health Organization; XQ, Xerostomia Questionnaire.

## 4. Discussion

Previous systematic reviews and meta-analyses have reported statistically significant yet modest clinical benefits associated with the adjunctive use of hyaluronic acid (HA) in periodontal therapy, including reductions in probing depth, gains in CAL, and improvements in BOP when combined with conventional non-surgical or surgical approaches [33,34]. However, these quantitative syntheses were largely based on studies conducted in systemically healthy or non-stratified populations, were characterized by short follow-up periods, and displayed substantial methodological heterogeneity. Consequently, their applicability to medically compromised patients whose inflammatory regulation and wound-healing capacity are intrinsically altered has remained limited [20].

The present narrative review specifically addresses this gap by integrating preclinical and clinical evidence related to HA use under conditions of systemic compromise. Across the reviewed studies, HA-based biomaterials demonstrated context-dependent biological effects, with clinical benefits most consistently observed in environments characterized by dysregulated inflammation, such as diabetes mellitus and oncologic disease [24,27,31,32]. It is important to note that the literature search was not restricted to specific systemic diagnoses. Rather, diabetes mellitus and oncology-related conditions emerged as the most consistently represented medically compromised states within the available evidence. This distribution likely reflects both their high clinical prevalence and their well-established association with impaired wound healing and dysregulated inflammatory responses in oral tissues, rather than an intentional narrowing of scope. These effects were predominantly expressed during the early phases of healing and included modest short-term improvements in periodontal parameters, reductions in local inflammatory biomarkers, accelerated epithelialization of extraction sockets, and improved patient-reported outcomes [24,27,28,33]. These findings align with established pathophysiological features of compromised healing, including exaggerated inflammatory responses, increased proteolytic activity, and delayed tissue repair [16,17], suggesting that HA may favorably modulate the early wound-healing microenvironment rather than directly induce regenerative processes.

Biological plausibility for these clinical observations is supported by translational and in vitro evidence. Cellular studies have demonstrated that HA enhances fibroblast migration and proliferation, promotes the expression of genes associated with regenerative and scarless healing phenotypes, and activates intracellular signaling pathways involved in tissue repair without inducing excessive inflammatory or matrix-degrading responses [35]. Complementary preclinical investigations conducted under uncontrolled hyperglycemic conditions further showed that cross-linked HA selectively attenuates inflammation-driven degradation processes, such as macrophage-mediated collagen membrane resorption, without impairing angiogenesis or tissue integration [12,13]. However, extrapolation from streptozotocin (STZ)-induced diabetic rat models to human clinical practice requires caution. Rodent models exhibit accelerated metabolic rates, differences in immune regulation, and faster collagen turnover compared with humans, which may influence biomaterial degradation kinetics and inflammatory responses. Together, these findings support a conceptual framework in which HA functions as a stabilizing functional biomaterial that buffers excessive inflammatory activity in compromised host environments rather than overriding physiological healing mechanisms [2,3].

In oral surgical applications, particularly extraction socket healing, clinical studies consistently reported enhanced early soft tissue closure and reduced postoperative morbidity following local HA application [25,26,27]. These effects are clinically relevant in medically compromised patients, for whom delayed epithelialization and postoperative discomfort may increase the risk of complications. However, evidence supporting sustained benefits on alveolar ridge preservation or long-term hard tissue stability beyond short-term follow-up (3–6 months) remains inconsistent. Future randomized trials incorporating ≥12-month radiographic follow-up are required to determine dimensional stability. HA should therefore be regarded as a supportive adjunct rather than a substitute for established regenerative or reconstructive strategies [36].

A similar pattern emerges in peri-implant therapy. Adjunctive HA use following mechanical debridement has been associated with short-term reductions in probing depths, inflammatory biomarkers, and transient microbiological shifts [28,29]. Nevertheless, current evidence does not support HA as a stand-alone therapy for peri-implantitis, and long-term effects on peri-implant bone stability remain insufficiently investigated [36]. Importantly, no clinical trials to date have specifically evaluated HA use in peri-implant therapy among medically compromised populations, representing a clinically relevant gap in the literature.

The most consistent clinical benefits of HA were observed in oncology-related oral complications. HA-based topical formulations were associated with reduced severity and duration of oral mucositis, improvement in xerostomia symptoms, and favorable patient-reported outcomes, without evidence of systemic adverse effects or interference with oncologic treatment modalities [30,31,32,33]. Given the limited therapeutic options available for mucositis management and its substantial impact on quality of life and treatment adherence, HA represents a valuable supportive care biomaterial in immunocompromised settings [34,36,37,38].

Several limitations should be acknowledged when interpreting these findings. This review follows a narrative design and does not include a formal risk-of-bias assessment or quantitative synthesis. The available literature is characterized by heterogeneity in HA formulations, including molecular weight, cross-linking, concentration, and application protocols, as well as predominantly short follow-up periods and relatively small sample sizes [12,34,36]. Furthermore, patients with poorly controlled or advanced systemic disease are underrepresented, potentially underestimating challenges encountered in routine clinical practice. Publication bias toward positive outcomes cannot be excluded.

## 5. Conclusions

Within the limitations of the available evidence, hyaluronic acid appears to function as a locally safe and biologically plausible functional biomaterial when used adjunctively in dental and periodontal therapies for medically compromised patients. Its clinical effects are primarily context-dependent and most evident during the early phases of healing, where modulation of the local inflammatory microenvironment may contribute to improved soft tissue healing and patient-reported outcomes.

Current data do not support a disease-modifying or stand-alone therapeutic role for hyaluronic acid in periodontal, peri-implant, or oral surgical applications. Rather, HA should be considered a supportive adjunct to established mechanical, surgical, and regenerative treatment protocols. Future research should prioritize well-designed, population-specific randomized clinical trials with longer follow-up periods, standardized HA formulations, and clear stratification of systemic disease severity to better define the role of HA-based biomaterials in compromised healing environments.

## Data Availability

No new data were created or analyzed in this study. Data sharing is not applicable to this article.

## Abbreviations

The following abbreviations are used in this manuscript:

BOP	Bleeding on Probing
CAL	Clinical Attachment Level
CBCT	Cone Beam Computed Tomography
CLHA	Cross-Linked Hyaluronic Acid
GBR	Guided Bone Regeneration
HA	Hyaluronic Acid
HSCT	Hematopoietic Stem Cell Transplantation
HMW	High-Molecular-Weight
HTN	Hypertension
LMW	Low-Molecular-Weight
MEDLINE	Medical Literature Analysis and Retrieval System Online
OMAS	Oral Mucositis Assessment Scale
PPD	Probing Pocket Depth
VAS	Visual Analog Scale
WHO	World Health Organization
XQ	Xerostomia Questionnaire
